# Blue light blind-spot stimulation upregulates b-wave and pattern ERG activity in myopes

**DOI:** 10.1038/s41598-021-88459-2

**Published:** 2021-04-29

**Authors:** Ana Amorim-de-Sousa, Tim Schilling, Paulo Fernandes, Yeshwanth Seshadri, Hamed Bahmani, José Manuel González-Méijome

**Affiliations:** 1 Clinical & Experimental Optometry Research Lab (CEORLab), Center of Physics (Optometry), School of Sciences, University of Minho, Gualtar, 4710-057 Braga, Portugal; 2 Dopavision GmbH, Berlin, Germany; 3 Department of Physiology of Cognitive Processes, Max Planck Institute for Biological Cybernetics, Tübingen, Germany; 4 Bernstein Center for Computational Neuroscience, Tübingen, Germany

**Keywords:** Translational research, Retina

## Abstract

Upregulation of retinal dopaminergic activity may be a target treatment for myopia progression. This study aimed to explore the viability of inducing changes in retinal electrical activity with short-wavelength light targeting melanopsin-expressing retinal ganglion cells (ipRGCs) passing through the optic nerve head. Fifteen healthy non-myopic or myopic young adults were recruited and underwent stimulation with blue light using a virtual reality headset device. Amplitudes and implicit times from photopic 3.0 b-wave and pattern electroretinogram (PERG) were measured at baseline and 10 and 20 min after stimulation. Relative changes were compared between non-myopes and myopes. The ERG b-wave amplitude was significantly larger 20 min after blind-spot stimulation compared to baseline (p < 0.001) and 10 min (p < 0.001) post-stimulation. PERG amplitude P50-N95 also showed a significant main effect for ‘Time after stimulation’ (p < 0.050). Implicit times showed no differences following blind-spot stimulation. PERG and b-wave changes after blind-spot stimulation were stronger in myopes than non-myopes. It is possible to induce significant changes in retinal electrical activity by stimulating ipRGCs axons at the optic nerve head with blue light. The results suggest that the changes in retinal electrical activity are located at the inner plexiform layer and are likely to involve the dopaminergic system.

## Introduction

Considering the increasing evidence of a rapid myopic trend in the younger cohorts of the global population, regulation of myopia progression has become a priority for the scientific community, eye care practitioners and policy makers. Different optical strategies have been developed and implemented over the past 10 to 15 years in an attempt to address this evolving global health concern^1^. Beyond the pattern of image formation, it is increasingly evident that the spectral composition and intensity of light are important to consider when attempting to interfere with myopia progression^2,3^. The contribution of intrinsically photosensitive retinal ganglion cells (ipRGCs) to different physiological functions has been elucidated in recent years and it is now possible to link ipRGCs to the modulation of the retinal activity^4^. Dopamine (DA) has been proposed as one of the neurotransmitters involved in the control of several physiological processes, including eye growth and refractive error development^5^. After the early discoveries of Stone et al. relating a decline in retinal dopamine with deprivation myopia, the potential involvement of DA in myopia development has been the subject of extensive review^6^. A previous study using electrophysiological techniques suggests that dopaminergic neurons receive excitatory input from synapses with ON bipolar cells in the inner plexiform layer (IPL)^7^.

Dopaminergic amacrine cells (DACs) are the main source of retinal dopamine, and are activated by rods, cones, and ipRGCs in response to light^8^. The activation of DA receptors, expressed by photoreceptors and amacrine cells, regulates gap junctions between different retinal elements and affects the amplitude of the electroretinogram (ERG) b-wave^9^. In fact, DACs can also be upregulated by stimulation of the ON bipolar circuit^10^. ON bipolar cells provide excitatory synaptic input to DACs, triggering dopamine release and the regulation of light responses in the inner retina. Li et al. injected chicken eyes with 6-OHDA which depletes DA from DAC. Authors showed small changes in dopaminergic pathways as measured with ERGs and oscillatory potentials. However, such changes were apparently strong enough to block development of deprivation myopia^11^. This is confirmed by the changes observed in the a- and b-waves of ERGs and oscillatory potentials^12^.

The effect of DA regulation on pattern electroretinogram (PERG) responses has also been observed in humans with Parkinson’s disease^13–15^. The progressive loss of retinal dopaminergic neurons and the consequent impact on dopamine regulation was strongly correlated with a latency delay and amplitude reduction observed in visual evoked potentials (VEPs) and PERGs of Parkinson’s disease patients when compared with healthy controls^16^. This decrease in PERG responses observed in Parkinson’s disease patients can be reversed with levodopa therapy^17^. Even patients in the early stages of Parkinson’s disease show the bioelectrical dysfunctions of the retina related to dopamine deficiency. This was evident by a reduction of mean amplitudes of several ERG tests, including a reduction in the photopic b-wave. In those patients, the application of dopamine antagonists induces a prolongation of the ERG a- and b-wave latency and a diminution in the b-wave amplitude^18^.

In clinical and experimental studies of human retinal function, the ERG b-wave is a measure that is commonly evaluated in research^19^. An important component of the ERG curve, the b-wave primarily reflects the post-synaptic retinal cells of the photoreceptors, namely the bipolar cells. Blocking the neurotransmission from photoreceptors to bipolar cells eliminates the b-wave^20,21^. In photopic ERG recordings, the b-wave is shaped by depolarizing ON-bipolar cells in the ascending phase and hyperpolarizing OFF-bipolar and horizontal cells in the descending phase, which pull the depolarization towards baseline^19^. The general function of retinal ganglion cells can be assessed using PERG recordings, where P50 and N95 peaks are of particular interest to researchers. In transient PERG, ON and OFF pathways contribute equally to the waveform. However, while P50 originates from both firing and non-firing of the pathways, N95 is suggested to be mainly related with firing activity^22^.

Melanopsin-expressing ipRGCs are a potential target system for a physiological enhancement of DA levels in the myopic eye, as they have been shown to project via their axon collaterals onto DACs^23^ and thereby modulate DA levels in response to light^24,25^.

The axons of ipRGCs pass through the optic nerve head, which corresponds to the blind-spot, and express melanopsin, as shown in rodents and humans^26–28^. Therefore, stimulation of the blind-spot with blue light overlapping with the sensitivity of melanopsin (around 480 nm^29^) could generate a retrograde effect of upregulation of DA secretion by DACs in the IPL^30^. Synaptic contact with bipolar cells at the same level might also be observed. This effect can be indirectly evaluated by analyzing the b-wave^31^.

Therefore, it was hypothesized that direct stimulation of the blind-spot with short-wavelength light would induce a retrograde effect on retinal ganglion cells, measurable by PERG, and that their interaction with bipolar cells in the IPL could be evaluated through the b-wave of full-field ERG (ffERG). We further hypothesized that such changes would be stronger in the myopic retina where a lack of DA could make them more sensitive to DA released in response to short-wavelength light^32^, and thus result in changes to the ERG response following blue light stimulation.

## Results

### First experiment: effect of blue light blind-spot stimulation in myopic eyes (b-wave light-adapted 3.0 ffERG)

**Figure 1:**
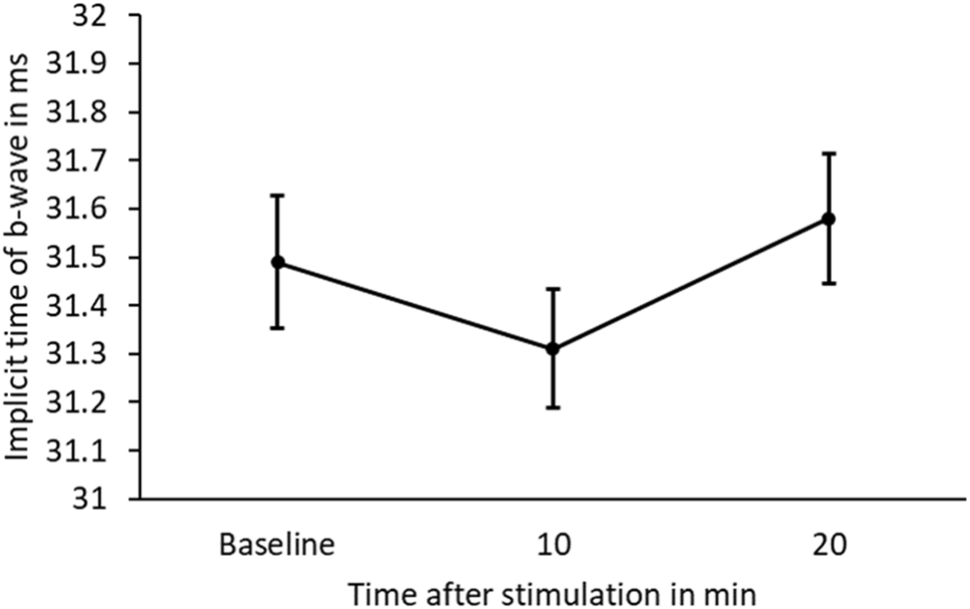
Mean and standard error of the mean (SEM) of implicit time of b-wave in ms for baseline and 10 and 20 min after blue light stimulation of the blind-spot in 10 myopes.

Statistical or clinically relevant differences were assessed in implicit time for all participants after blind-spot stimulation. Repeated measures ANOVA did not show a significant difference (p = 0.157). The results are shown as mean and standard error of mean (SEM) values in Table 1 and Fig. 1.

**Table 1:** Implicit times of photopic ffERG in myopes.

	Baseline [ms]	10 min [ms]	20 min [ms]
**b-wave n = 10**			
Mean	31.5	31.3	31.6
Std. error mean	0.276	0.245	0.269

**Figure 2:**
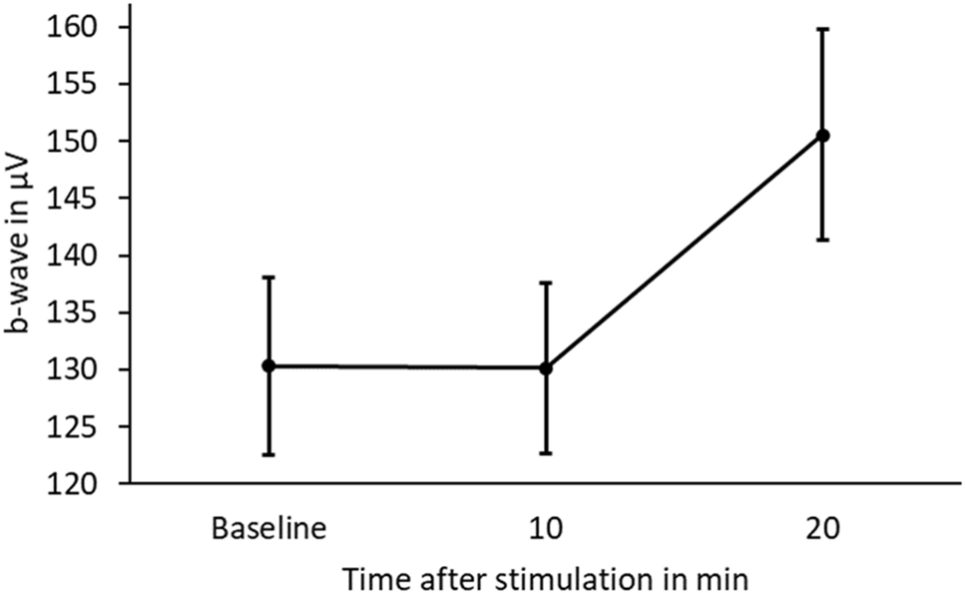
Mean and standard error of the mean (SEM) of b-wave in µV for baseline and 10 and 20 min after blue light stimulation of the blind-spot in 10 myopes.

When assessing the changes in amplitude of the b-wave after stimulation, a repeated measures ANOVA revealed a significant main effect for ‘Time after stimulation’ (p < 0.001). Tukey-corrected post-hoc tests showed that the b-wave was significantly larger after 20 min compared to baseline (p < 0.001) and 10 min (p < 0.001) after stimulation of the blind-spot with blue light (see Fig. 2). No significant difference was observed between baseline and the 10 min condition (p = 0.990).

### Second experiment: is the previous effect observed in PERG?

**Figure 3:**
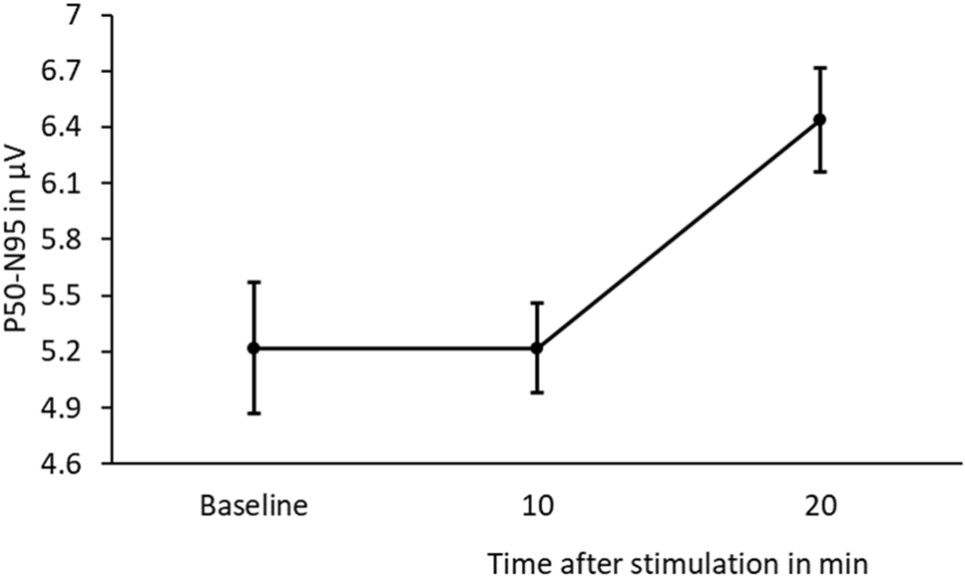
Mean and standard error of the mean (SEM) of PERG P50-N95 in µV of baseline and 10 and 20 min after blue light stimulation of the blind-spot in 5 myopes.

In PERG P50-N95, a repeated measures ANOVA revealed a significant main effect for ‘Time after stimulation’ (p < 0.050). Post-hoc tests corrected with Tukey showed that P50-N95 was significantly larger 20 min after blind-spot stimulation compared to baseline (p < 0.050) and the 10 min condition (p < 0.050), such as previously observed in the b-wave amplitude (see Fig. 3).

**Figure 4:**
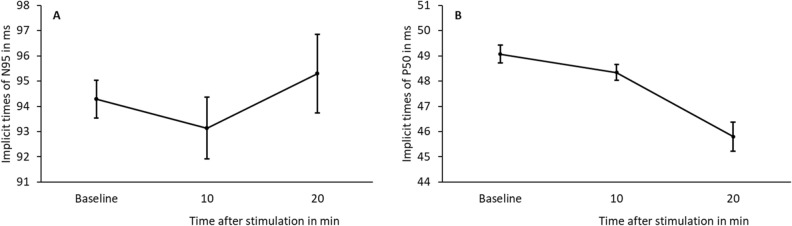
Mean and standard error of the mean (SEM) of implicit time of PERG P50 (**A**) and N95 (**B**) in ms of baseline and 10 and 20 min after blue light stimulation of the blind-spot in 5 myopes.

Once again, implicit time did not show any significant differences after blind-spot stimulation in a repeated ANOVA for P50 (p = 0.109) and N95 (p = 0.741). See mean and SEM in Table 2 and Fig. 4A,B (P50 and N95 implicit time, respectively).

**Table 2:** Implicit times of PERG in myopes.

	Baseline [ms]	10 min [ms]	20 min [ms]
**P50 n = 5**			
Mean	49.1	48.3	45.8
Std. error mean	0.697	0.628	1.17
**N95 n = 5**			
Mean	94.3	93.1	95.3
Std. error mean	1.50	2.43	3.11

### Third experiment: comparison between myopes and non-myopes

**Figure 5:**
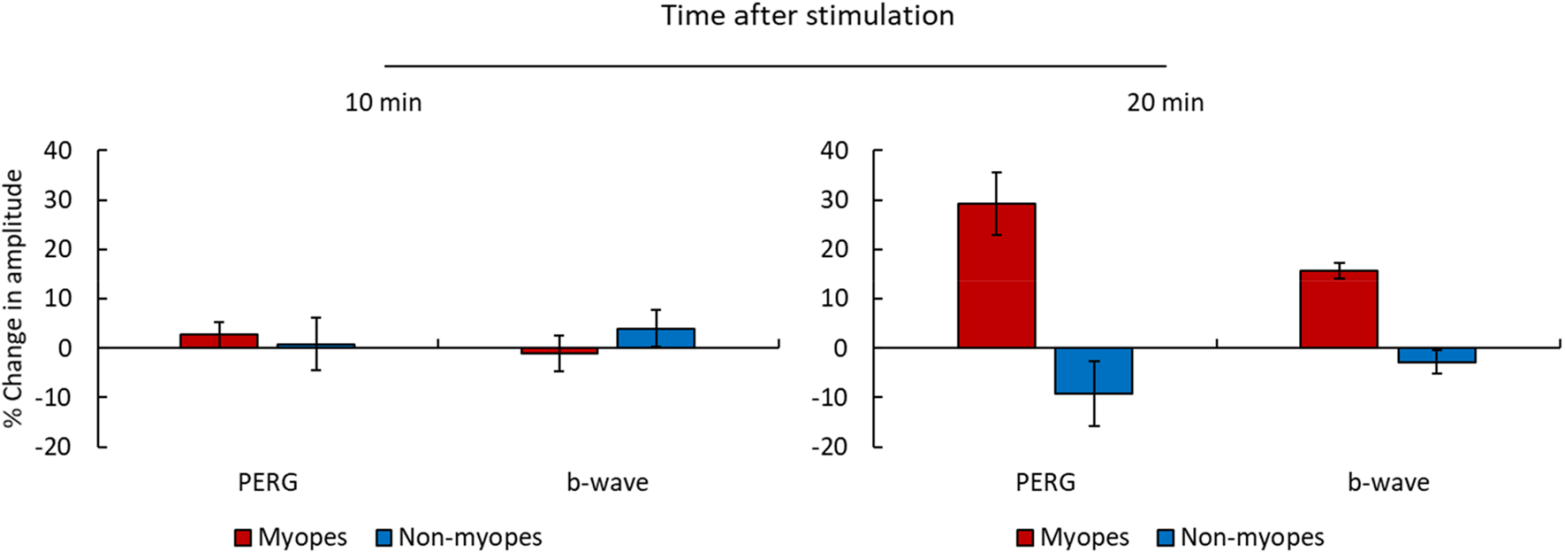
Mean and standard error of the mean (SEM) of change in ERG amplitude relative to baseline in 5 myopes and 5 non-myopes for b-wave and PERG P50-N95 at 10 min (left) and 20 min (right) after stimulation.

After blind-spot stimulation an increase in both the PERG and b-wave was observed in myopes but not in non-myopes. The independent one-sided t-test showed a significantly larger change in amplitude in myopes compared to non-myopes in the PERG P50-N95 (p < 0.050) and in the b-wave (p < 0.010), 20 min after blind-spot stimulation with blue light (Fig. 5). No significant difference was found after 10 min of blind-spot stimulation in the PERG P50-N95 (p = 0.510) or in the b-wave (p = 0.680).

## Discussion

To the best of our knowledge, the present study is the first evaluating the effect of stimulating the blind-spot with blue light using a virtual reality (VR) system on the electrophysiological response of the human retina. The results of this study have revealed two important points. First, that the level of retinal activity measured by photopic 3.0 of ffERG and PERG, potentially related to the dopaminergic path, can be increased in myopic eyes after blue light stimulation of the blind-spot. This may reflect a retrograde feedback effect of the ipRGCs at the level of the IPL where they connect with amacrine and bipolar cells as shown by the PERG and b-wave response. Second, the effect we have shown was observed in myopes and not in non-myopes, and therefore this stimulation technology may have major implications for future myopia control.

The amplitude of both the b-wave and PERG responses only increased in myopic eyes 20 min after the blind-spot was stimulated with blue light.

Although statistical analyses did not indicate a significant change in the implicit time following blind-spot stimulation, the small, but apparent, acceleration of the P50 peak should not be overlooked as it suggests that blue light stimulation may induce a faster response of ON and OFF pathways. In fact, these non-significant trends in implicit time are worth investigating in future studies with a greater number of participants and can be used as informative average of expected values for similar studies.

Myopic eyes have been shown to have lower levels of dopaminergic activity within the retina and susceptibility to form deprivation myopia in animal models was higher when the DOPAC/ DA ratio was lower^33^. This suggests that lower metabolic activity involving DA is associated with a stronger predisposition to develop higher degrees of myopia^33^.

Based on the simultaneous upregulation of the PERG and b-wave observed in the present study in myopic eyes, we can advance with a proposed mechanism that confirms the hypothesis raised in this study. Connectivity between ipRGCs and DACs has been established at the level of the IPL^34^. Zhou et al. have proposed a mechanism in which bright flickering light that stimulates the ON pathway and ipRGCs can alter DA synthesis and release^6^. In the present study a flickering blue light within the maximum range of sensitivity of melanopsin-containing ipRGCs has been used. A relationship between ipRGCs and DACs has been established as both are driven by ON bipolar cells stratifying in the outermost IPL^10^. Therefore, it is plausible that the activation of ipRGCs, as measured by the PERG, could be responsible for the observed b-wave changes. Such changes may reflect the upregulation of DA as a result of the link between ipRGCs and DACs at the level of the IPL. The protective role of the activation of the ON channel in myopia has been documented in several animal models^35^. The present results open the door to the use of this intervention approach in humans.

Additional research has found that exposure to bright light during outdoor activities is effective at delaying the onset of myopia and that this effect may be related to higher DA activity^36,37^. Therefore, the results of the current study point to the potential of blind-spot stimulation with short-wavelength light to elicit a retrograde effect in the IPL. This retrograde effect may be reflected in the increased PERG amplitude after blind-spot stimulation that is mirrored in the b-wave amplitude of myopes, independent of the degree of change created by the blue light stimulus at each retinal layer. Similar b-wave behavior for different levels of change in PERG activity could indicate a binary gate. In such a case the increase in b-wave activity does not increase linearly with the degree of PERG amplitude change. Rather, the b-wave increases to a similar level irrespective of the PERG response once the PERG is upregulated. This is true even at different levels across individuals, as the b-wave upregulation is similar for different participants.

An intriguing question from the present study arises from the fact that unlike myopes, non-myopes showed fairly stable PERG and b-wave activity over the course of the first experiment. A previous study with guinea pigs showed that apomorphine, a non-selective DA receptor agonist, did not change eye growth in guinea pigs with normal vision^38^. A possible explanation would be that non-myopes already have high levels of DA activity, which could limit the improvement of retinal activity and eye growth, whereas DA receptors in myopes may show a higher affinity to DA production/release by exogenous factors. Feldkaemper and Schaeffel also suggested that DA receptors may be involved in some regulatory mechanisms, although there is no agreement between studies on the topic^39^. Another interesting question to address in the future is if the induced effect influences the whole retina, or some specific areas preferentially. Stimulation of the blind-spot with blue light might activate melanopsin-expressing ganglion cells. The blind-spot corresponds to the optic nerve head where the axons of ipRGCs pass through and express melanopsin, as shown in rats^26^. The dendrites of these cells were found to co-stratify^23^ and signal with DACs (peak density at 2.5–3 mm eccentricity)^40^ in a retrograde manner^41,42^, suggesting that photosensitive retinal ganglion cells might play a role in retinal DA regulation. However, Munteanu et. al. found a light-dependent development and functionality of DACs and DA levels with no influence of photosensitive ganglion cells in mice. Alternatively, a major contribution of the rod-pathway to the DA regulatory mechanism was suggested^43^.

In this study the blind-spot, which corresponds to the optic nerve head, was stimulated with blue-light via VR system. However, the optic nerve head can be stimulated also via silent substitution technology^44^. Eye movements could be recorded during stimulation in future experiments as soon as the technical limitations of an eye-tracker in the VR system are solved and allow a precise fixation determination.

A potential limitation of the present study is that trials have been done in young adults aged 18 to 25 years, while the target population for myopia control treatments is younger^45^. Typically, children would be between the ages of 6 and 12 years, and continue their treatments until 18 years or older^46,47^. The need for information concerning the levels of DA in children’s retinas, particularly in those at risk of developing myopia, has been raised by Zhou et al.^6^. Several ERG studies investigating retinal function point to a reduction in the retinal activity of myopes compared to emmetropes^48,49^. In a previous study with myopic children from the ATOM 2 study, no association was found between the ffERG amplitude and axial length. Rather, an association between retinal sensitivity and baseline axial length was identified, suggesting a loss of retinal sensitivity prior to the typical amplitude reduction found in myopic adults^50^. Li et al. observed changes over one year in refraction, axial elongation, and retinal activity (global-flash mfERG) in emmetropic children 6–9 years old. After one year they registered an axial elongation around 0.37 mm with a mean spherical refraction of − 0.55 D in most children. They also noted that children who became myopes already presented a subclinical reduction of central inner retinal function prior to myopia development compared to those who remained emmetropes^51^.

Another potential limitation of the present study is that measurements were not taken at the same time of the day for all participants. However, for each participant, PERG and b-wave recordings were obtained within the same period of the day in order to avoid the effect of circadian changes when comparing the retinal response^4^. We conducted a brief analysis to verify whether participants measured during the morning differed from those measured during the afternoon (data not shown in this study). Despite differences in absolute values, an increase in retinal activity after 20 min was observed independently of the time of the day.

The assumption that IPL is potentially mediated by DA release after blind-spot stimulation is consistent with the structure of the ON and OFF pathway circuits in the mammalian retina at the level of the IPL and could be mediated by ON bipolar cells synapsing with ipRGCs and amacrine cells^4^. Therefore, based on the present results we can hypothesize that the upregulation of retinal electrical activity could be related to an upregulation of DA release. Interestingly, this effect is quite selective of myopic eyes, rather than emmetropic eyes, which showed consistent activity before and after stimulation. This suggests that the blue light stimulus only produced an improvement in myopes, where retinal activity may be compromised due to some anatomical or physiological changes, while normal eyes did not experience any effect of the treatment. In a similar way, previous studies reported improvements in the retinal activity of diabetic animals after administrating treatment, while in normal eyes no influence of the treatment was observed^52–54^.

This raises the possibility to interfere with the development of refractive error by stimulating DA release in myopic patients. The approach presented in this study demonstrates that this can be achieved without direct exposure of the retina to short-wavelength light, by instead stimulating the system through the blind-spot. Another question raised from this study is if treatments that intend to stimulate DA release in the retina might be effective at delaying myopia onset, slowing its progression, or if both prophylactic and therapeutic effects might be possible. This is certainly an important question to answer in future clinical trials.

In conclusion, this study showed that following blind-spot stimulation with blue light the amplitudes of the PERG’s P50-N95 and ERG’s b-wave are increased after 20 min in myopes. These changes in amplitude are larger in myopes than in non-myopes. Therefore, the results presented in this study confirm and reinforce the hypothesis that stimulation of the blind-spot with short-wavelength light matching the sensitivity of melanopsin in ipRGCs elicits increased activity in retinal ganglion cells as measured with the PERG technique. This also activates what seems to be a retrograde effect in the IPL that might involve an upregulation of DA release, by direct contact with DACs, the increased ON bipolar circuit observed in the b-wave, or through both mechanisms.

## Methods

This was an exploratory study conducted by the Clinical and Experimental Optometry Research Laboratory at the University of Minho (Braga, Portugal). The study was divided into three experiments. The first experiment aimed to observe the effect of stimulating the blind-spot with blue light on the b-wave recorded with the light-adapted 3.0 protocol of the ffERG in a myopic population. The purpose of the second experiment was to verify if the previous effect was also present in the ganglionic layer of myopic participants by recording PERG responses. The third experiment tested the hypothesis that after blue light stimulation in the blind-spot myopes would show a larger change in amplitude than non-myopes.

### Participants

For the purpose of the study, young and healthy participants without any history of ocular or systemic disease were recruited based on the following inclusive criteria: age between 18 and 30 years, manifest refractions between + 0.50 Diopters (D) and − 4.00 D, astigmatism below 1.00 D, and visual acuity of at least 0.0 logMAR with habitual correction. This was to avoid any influences on the ERG response due to structural changes in the retina caused by axial elongation^55,56^.

In the first experiment, ten myopic participants (− 1.90 ± 1.19 D) with mean age of 25.0 ± 4.6 years were recruited for light-adapted 3.0 b-wave ERG measurements. Thereafter, five of those myopes were randomly selected (mean age of 24.8 ± 5.8 years; mean spherical equivalent of − 1.90 ± 1.40 D) for PERG recordings (second experiment), considering that the b-wave effect of the first experiment was observed in all participants. For the third experiment, the b-wave and PERG recording of the same five myopic participants were compared with 5 non-myopic participants (mean age of 25.2 ± 3.7; mean spherical equivalent of 0.08 ± 0.23D).

### Protocol

The protocol of the three experiments followed the principles of the Declaration of Helsinki and was approved by the Ethics Committee for Health and Life Sciences of the University of Minho. All participants provided written informed consent. Measurements for all experiments were taken between 10:00 a.m. and 5:00 p.m. to avoid the influence of DA on the circadian cycle, and participants had no caffeine or nicotine consumption at least 5 h before each measurement. The full protocol included VR calibration of each individual’s blind-spot position before ERG electrode placement; baseline ERG measurement followed by blind-spot stimulation^44,57,58^; and further ERG acquisitions thereafter. The b-wave and PERG protocols for each participant were performed on different days, but during the same period (morning or afternoon).

**Figure 6:**
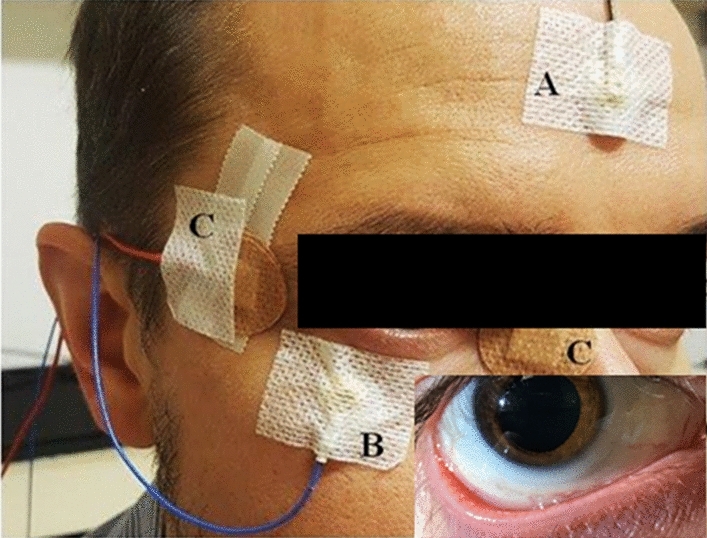
Illustrative position of the electrodes for ERG measurements. (**A**) Gold-cup ground electrode; (**B**) God-cup reference electrode; (**C**) DTL active electrode. For illustration purpose, small picture insert shows the position of the DTL electrode in the conjunctival area.

The RETI-port/scan21 (Roland Consult, Wiesbaden, Germany) was used for the electrophysiological recordings following the ISCEV guidelines^59,60^. Before placing the electrodes, the skin was cleansed with an abrasive gel. Next, gold-cup reference and ground electrodes and active DTL-plus electrodes (Dawson-Trick-Litzkow) were placed (Fig. 6). Impedance was checked before each measurement and recordings only taken when it was smaller than 5 kOhm. All ERG measurements were recorded binocularly. To maintain a consistent degree of adaptability between participants and measurements prior to each b-wave recording participants underwent 10 min of light adaptation in the ERG Ganzfeld at a constant background luminous intensity of 30 cd/m^2^, measured with a luminance meter (LS-150, Konica Minolta, Osaka). Before each PERG measurement, participants had a 5-min break. Both ERG protocols took place in an isolated room with a Faraday cage, under ambient lighting of approximately 400 lx, measured using an illuminance meter (T-10A, Konica Minolta, Osaka). To ensure the environment conditions, before each protocol we repeatedly measure the illuminance at the point of examination (at 1 m from the display at eye’s level in PERG, and at the measure position of Ganzfeld in b-wave). ERG responses were recorded before (baseline), and 10 and 20 min after 1-min stimulus exposure in order to assess the possible differences on the effect of blind-spot stimulation with blue light.

Although ISCEV ERG protocols require a fully dilated and stable pupil size it is unknown to what extent topical mydriatics might influence the response of ipRGCs to blue light stimulation. According to Mojumder and Wensel, topical administration of atropine and phenylephrine together, but not separately, leads to a slow, dramatic enhancement of a- and b-waves by an unknown mechanism independent of pupil dilation^61^. To account for this finding and to work under normal physiological conditions of pupil size (a critical factor in determining the effectiveness of the treatment) measures were recorded under non-dilated conditions. The pupil size is obtained 3 times per patient to obtain an average value. Each measurement session takes several seconds, which allow the pupil to fluctuate within the physiological rhythm. Therefore, the value obtained should be understood not only as an instantaneous measurement but an average value within the normal fluctuations of the pupil. Pupil size was checked with an infrared pupilometer (VIP-200, NeurOptics, California) before and after all ERG measurements to ensure that luminance conditions were stable, with minimal fluctuations within physiological terms, without adverse impact for the consistency of the measurements.

### ERG tests

In this study, the light-adapted 3.0 ERG test of ffERG was recorded in all participants and the PERG was recorded only in those participants included in the second and third experiments. The two ERG methodologies assess the electrophysiological response of different cellular groups in the retina (cones and bipolar cells^59^, and ganglion cells^60^, respectively) of both eyes.

**Figure 7:**
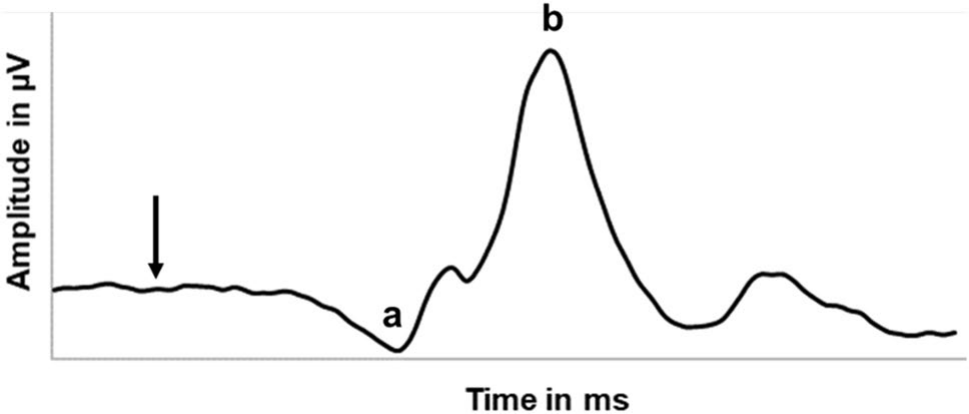
Illustrative diagram of the basic ERG output of the light-adapted 3.0 ffERG with its 2 main curves: a-wave and b-wave. The arrow indicates the stimulus flash.

For the light-adapted 3.0 ERG test we used a sequence of five single-flashes of white light (3.0 cd.s/m^2^) generated in a Ganzfeld stimulator against a light white background (30 cd/m^2^), with a stimulus rate of 0.625 Hz and a recording bandpass filter of 1–300 Hz. The resulting wave response was a photopic single-flash cone response, reproducing discernible a- and b-waves^59^, similar to the one represented in Fig. 7.

**Figure 8:**
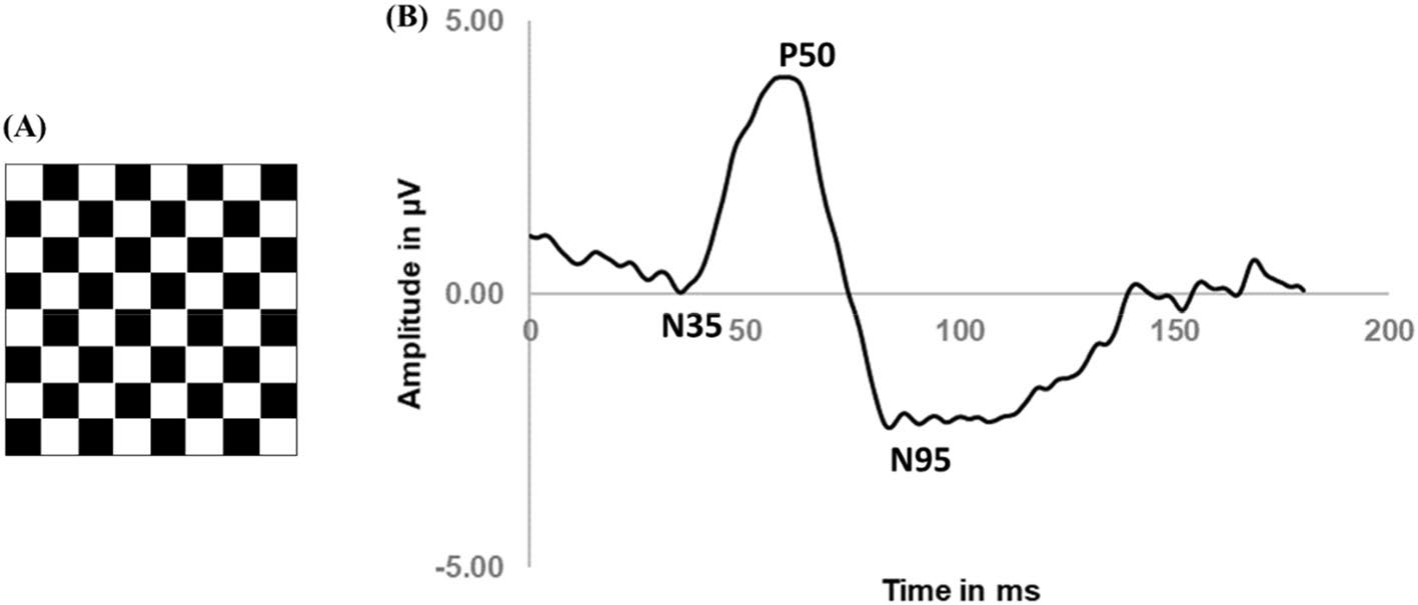
(**A**) Stimulus used for recording ganglion cell electrical activity with PERG methodology. The black and white checks alternate from black-to-white and white-to-black at a regular frequency, with a nearly 100% luminance contrast between checks. (**B**) Typical PERG wave response (example from one of the participants).

For the PERG evaluation, the transient PERG protocol was used. This protocol detects the retinal activity in response to the reversal of black and white squares of a checkerboard stimulus (Fig. 8A). In this study, the pattern stimulus was generated on an LCD monitor (ProLite B1980SD, iiyama) with a frame rate of 60 Hz, covering a field size of 15º at an observation distance of 1 m. The black and white reversing checkerboard was presented with a check size of 0.8º for a transient reversal of 1.53 rev/s, with a mean illuminance of 152.64 ± 0.64 lx (Illuminance meter T-10A, Konica Minolta, Osaka). The mean luminance of the black and white checks was 1.47 ± 0.06 cd/m^2^ and 220.32 ± 1.23 cd/m^2^, respectively (Luminance meter LS-150, Konica Minolta, Osaka). Participants were asked to maintain their fixation on a red *X* located at the corner of the four central squares. The signals were amplified and filtered (first-order bandpass 5–50 Hz). Sweep length was 180 ms (sample freq. 2.84 Hz) and 200 sweeps used for averaging. PERG recordings result in a wave response similar to the one represented in Fig. 8B. In general, the positive peak (P50) is produced by the retinal ganglion cells and other inner cells, while N95 is generated almost exclusively by retinal ganglion cells^60^.

### Blind-spot stimulation

The stimulation of the optic nerve head with blue light was performed with a proprietary application (VR stimulation application, Dopavision GmbH, Germany) for the Android smartphone Samsung Galaxy S7 (Samsung, Seoul, South Korea) with a Super AMOLED display (5.1 in., 1440 × 2560 pixels) inserted in a VR system (Trust International B.V., Dordrecht, Netherlands) called VR box. Participants used this app by placing the smartphone inside the VR box. For setup calibration and to ensure that the blue light stimulus fell onto the blind-spot, participants were instructed to use the built-in controls to adjust the position of a red disc until it was no longer visible while looking at an ABC fixation target, which is a combination of bullseye and crosshair that has been shown to have high fixation stability^62^. Participants had to match the stimulus size of less than 3° on the smartphone display for each eye separately until they did not see the blind-spot stimulus, thereby ensuring that the stimulus fell on the optic nerve head while looking to the fixation target. The stimulus presentation consisted of a blue stimulus (450 nm peak wavelength, which is within the maximum relative sensitivity of melanopsin) at the calibrated blind-spot, with a flicker frequency of 15 Hz and luminance of 14.74 ± 1.11 cd/m^2^, and a black background. Flicker at 15 Hz was used because low temporal frequencies should be avoided for its negative effects on myopia development, as demonstrated in guinea pigs and chickens^35,63^. Furthermore, it has been shown that 1 h of either 2 or 20 Hz flickering illumination results in a reduction of DOPA, whereas in between temporal frequencies such as 10 Hz showed the opposite^64^. In the present study the exposure time to the stimulus was 1 min. Participants were instructed to carefully keep fixation on the fixation target and not to move their eyes during the stimulation period.

### Data analysis

Data analyses were conducted on the implicit time and amplitude of the b-waves of the light-adapted curve ERG cone response and of P50-N95 of the PERG. For the comparison between myopes and non-myopes, ERG amplitudes were normalized to the baseline. Although both ERG measurements and blind-spot stimulation was performed binocularly, only ERG responses of one eye for each participant were considered. The eye was selected based on the blind-spot calibration. The eye with the calibration closest to the average calibration position was selected in order to reduce potentially large individual differences in blind-spot position. This also had the advantage of reducing the impact of any calibrated positions that were unintentionally inexact.

### Statistical analysis

The sample sizes of the three experiments ensure 80% power for statistical analysis. Repeated measures ANOVAs for the factor ‘Time after stimulation’ with Tukey-corrected post-hoc tests were conducted^65,66^ for amplitude and corresponding implicit times separately. To test the hypothesis, an independent one-sided t-test was performed on change in amplitude. For statistical analysis JAMOVI (1.1.9.0, jamovi project, 2019)^67^ was used.

## Data Availability

The datasets generated during and/or analysed during the current study are available from the corresponding author on reasonable request.
